# Genomic and enzymatic signatures underlying lifestyle diversity in Xanthomonas arboricola

**DOI:** 10.1099/mgen.0.001782

**Published:** 2026-07-16

**Authors:** Sara Cuesta-Morrondo, Salvador Sastre, Jerson Garita-Cambronero, Jaime Cubero

**Affiliations:** 1 Instituto Nacional de Investigación y Tecnología Agraria y Alimentaria (INIA), Consejo Superior de Investigaciones Científicas (CSIC), Madrid, Spain; 2 Instituto Tecnológico Agrario de Castilla y León (ITACyL), Castilla y León, Spain

**Keywords:** commensal, hazelnut, plant pathogenic bacteria, *Prunus* spp., walnut, xanthomonads

## Abstract

*Xanthomonas arboricola* is a plant-associated bacterial species that comprises the pathovars pruni (*Xap*), juglandis (*Xaj*), corylina (*Xac*), which are of high economic concern; and the pathovars arracaciae, celebensis, fragariae and zantedeschiae, which are less relevant, meaning they have a lower economic impact, are less widely distributed, or cause less severe diseases in their host plants. Moreover, the species also includes strains without pathovar affiliation. The pathogenicity of the less economically relevant pathovars as well as the non-affiliated strains has been debated. The present study analysed all the *X. arboricola* genomes deposited in GenBank by March 2025, focusing on type III secretion system (T3SS), type III effectors (T3Es), cell wall-degrading enzymes (CWDE), amylases and hydrolytic activity.

The average nucleotide identity analysis showed that strains belonging to the three main pathovars clustered according to their pathovar designation, harbouring both T3SS and a large T3Es repertoire. However, strains belonging to pathovars of lower economic relevance or with no pathovar affiliation were clustered into two groups, G1 and G2. Remarkably, strains belonging to these less relevant pathovars were scattered along these two groups, exhibiting notable genetic diversity. Moreover, strains in G1 generally displayed T3SS but a limited set of T3Es, whereas strains in G2 lacked a T3SS and exhibited an even more reduced T3Es repertoire. Regarding CWDE, differences in the amount and profiles of cellulolytic, hemicellulolytic and pectinolytic enzymes were observed. While *Xap* and *Xaj* possessed the highest counts of hemicellulolytic enzymes, strains in G1 and G2 harboured the highest numbers of cellulolytic and pectinolytic enzymes. Moreover, hydrolytic activities were assayed in a set of strains comprising the three main pathovars and G1 and G2 clusters. It was demonstrated that G1 and G2 strains caused soft rot in pepper fruits, while the main pathovars did not. Furthermore, all the *X. arboricola* strains analysed hydrolysed starch, except for *Xap*, which also harboured a reduced set of amylolytic enzymes. The doubtful, atypical or weak pathogenicity, the genomic diversity, the limited content of T3Es, together with the soft rot capacity, the higher counts of cellulolytic and pectinolytic enzymes and the literature, suggest that strains in G1 and G2 would probably be commensal or saprophytic strains that may behave as opportunistic pathogens under certain conditions.

**Impact Statement**

*Xanthomonas arboricola* is a plant-associated bacterial species containing economically important pathovars (pruni, juglandis, corylina) plus less virulent and non-affiliated strains. Here, we present the most comprehensive genomic analysis of all *X. arboricola* genomes released up to date (March 2025) in which all key pathogenicity factors, such as type III secretion system (T3SS), type III effectors (T3Es), cell wall-degrading enzymes and hydrolytic activity, were examined. Our results indicate clear genetic grouping: major pathovars carry the T3SS and an extensive arsenal of T3Es, whereas strains that clustered into two groups (G1 and G2) display high-genomic diversity, low content of T3Es and, in the case of G2, no T3SS presence. Remarkably, the G1 and G2 strains induced soft rot in pepper fruits, indicating an opportunistic rather than a typical pathogen-host relationship. These findings call into question prevailing concepts of species pathogenicity, with many such strains potentially being commensal or saprophytic in nature, only displaying opportunism. In this sense, it contributes to research on *X. arboricola* ecology and evolution with a wide range of implications for taxonomy, phytosanitary risk and management options in plant crops, but also for the theoretical basis concerning host-microbe interactions in agricultural systems.

## Data Summary

The authors confirm all supporting data, code and protocols have been provided within the article or through supplementary data files. The genomic data used in the study were previously published and are available from the National Center for Biotechnology Information (NCBI). All accession numbers are provided in Table S1 (available in the online Supplementary Material).

## Introduction

*Xanthomonas arboricola* is a species of Gram-negative, plant-associated bacteria found in diverse agricultural and non-agricultural niches. This species encompasses seven pathovars, as well as other strains without pathovar affiliation. The three pathovars with the greatest agricultural and economic impact are pathovar (pv.) pruni, pv. juglandis and pv. corylina. These pathovars are considered highly relevant because they cause severe diseases in economically important crops, lead to significant yield losses and have consequently been classified as regulated or quarantine pests in several countries. In contrast, pvs. arracaciae, celebensis, fragariae and zantedeschiae are considered less relevant, as they are associated with crops of lower economic importance, show a more limited distribution, or generally cause less severe disease outbreaks [1,2].

*X. arboricola* pv. pruni (*Xap*) is a phytopathogenic bacterium that causes bacterial spot on almond (*Prunus dulcis*), several stone fruit trees, such as *Prunus salicina*, *Prunus persica*, *Prunus domestica*, *Prunus armeniaca* and ornamental trees like *Prunus laurocerasus*. This disease is characterized by symptoms, including leaf spots and shot-holes, sunken necrotic lesions on fruits, cankers on twigs and trunks and, in severe cases, reduced production, defoliation and premature fruit dropping. Although bacterial spot of *Prunus* spp. was first reported in the USA in the twentieth century, it is now worldwide distributed, spreading to new regions every year [3,4].

*X. arboricola* pv. juglandis (*Xaj*) infects *Juglans* spp., causing three different diseases: walnut bacterial blight (WBB), vertical oozing canker (VOC) and brown apical necrosis (BAN). WBB is a common disease of walnut and is characterized by necrotic spots on leaves, twigs and fruits. VOC causes vertical cankers with brown exudates on branches and trunks. Finally, BAN, caused by *Xaj* in association with fungal pathogens, is characterized by apical necrotic lesions near the blossom end of the fruit. *Xaj* mainly infects Persian walnut (*Juglans regia*), although other *Juglans* spp. have also been reported as hosts, including *Juglans nigra*, *Juglans californica*, *Juglans hindsii* and *Juglans cinerea*, among others [2].

*X. arboricola* pv. corylina (*Xac*) causes bacterial blight on *Corylus* spp., which is characterized by cankers in stems and shoots, water-soaked and necrotic angular lesions on leaves and necrotic lesions on the shell of the fruits. The principal host of *Xac* is *Corylus avellana* (hazel), but it also infects other *Corylus* spp., such as *Corylus colurna, Corylus pontica* and *Corylus maxima* [2].

In addition to these three economically relevant pathovars, *X. arboricola* comprises other pathovars that have been isolated from both agricultural and non-agricultural sources. Among the agricultural-related pathovars are *X. arboricola* pv. arracaciae, isolated from *Arracacia xanthorrhiza*, pv. celebensis, isolated from banana and pv. fragariae, isolated from strawberry, although the pathogenic status of the latter remains ambiguous [1,2, 5,7]. Moreover, *X. arboricola* pv. zantedeschiae was isolated from the ornamental species *Zantedeschia aethiopica* [1,5]. These pathovars could be considered as less relevant, on account of the rarity of reports of occurrence [5,8].

*X. arboricola* also comprises non-pathogenic or rarely reported, low-pathogenic, saprophytic, commensal and opportunistic strains which have been isolated from a variety of plants and, according to the GenBank database, from environmental samples, such as soil and rainwater [1,5] (Table S1). Garita-Cambronero *et al*. described *X. arboricola* strain CITA 44, which was isolated in Spain from asymptomatic leaves of *Prunus mahaleb* (rootstock Santa Lucía SL-64) [9]. Moreover, they also identified strains CITA 14 and CITA 124, which were isolated from asymptomatic peach trees in Spain [10]. These strains were referred to as *Xap* look-alikes, as they were isolated from *Prunus* spp., which led to their initial misidentification as pv. pruni. Inoculation of these strains in different plant species caused the appearance of minor symptoms in some cases; however, their bacterial populations remained low after the incubation time compared to *Xap* strains, indicating that they were not pathogenic [11,12]. Other * X. arboricola* strains with no pathovar affiliation have been isolated from symptomatic plants worldwide, such as the strain 3004, which was isolated in Russia from barley leaves with streak symptoms; strains 1311a and 1314c, which were isolated in Poland from blueberry plants with bacterial leaf blight; or strains BC2526, BC2527, BC2528 and BC2529, which were isolated in Korea from bell pepper displaying leaf spot symptoms [13,15]. Given that some of these reports date back over a decade and that, based on the literature reviewed, these diseases have not re-emerged nor been classified as significant threats by the EPPO or other regulatory bodies, it is reasonable to infer that these bacterial strains exhibit low pathogenic potential.

Several virulence factors have been proposed in *X. arboricola* to be fundamental for disease development. Among them, the most notable is the type III secretion system (T3SS), which secretes type III effectors (T3Es) addressed to manipulate the host metabolism and defences [16]. Other relevant virulence factors include adhesins, chemotactic proteins, methyl-accepting proteins, TonB-dependent transporters and cell wall-degrading enzymes (CWDE), among others [17]. CWDE are key virulence factors in many phytopathogenic bacteria and mainly include cellulolytic, hemicellulolytic and pectinolytic enzymes. These enzymes break down plant cell wall components, facilitating tissue invasion, symptom development and nutrient acquisition and act in coordination with other virulence systems [18]. Many of these CWDE are secreted by the type II secretion system (T2SS) Xps, while some have been observed to be secreted via outer membrane vesicles (OMVs) [16,19]. Furthermore, *Xanthomonas* secretes other enzymes targeted to degrade other plant tissues, such as the amylolytic enzymes, which hydrolyse the starch in fruits, seeds and stems and may play a role in virulence [20,23]. Extracellular enzymes are thought to contribute to disease progression, facilitating bacterial spread into uninfected plant tissues or breaking down plant polymers for nutrient acquisition [23].

Although the pathogenicity traits and diversity of some *X. arboricola* strains have been examined from a genomic perspective and the genomes of some strains have been sequenced, these analyses were confined to a limited number of strains [5,24,31]. The present study aims to analyse all the *X. arboricola* genomic sequences deposited in GenBank until March 2025, including pathogenic and non- or low-pathogenic members. The goal is to explore host interactions across the entire species, with a particular focus on the T3SS and its effectors, as well as hydrolytic enzymes that could enable low-virulence strains to exploit plant-derived nutrients.

## Methods

### Genomic analysis of all *X. arboricola* strains in GenBank

The *X. arboricola* genomes deposited in GenBank by March 2025 (Table S1) were analysed. Average nucleotide identity (ANI) among the genomes was calculated using FastANI v1.3 [32] implemented within the Galaxy platform (https://usegalaxy.eu/) [33] and, afterwards, an UPGMA dendrogram was created with mega version 11.0.13 [34] and iTOL v7 [35]. The ANI results were also represented as a heatmap using matplotlib [36]. Plasmids were reconstructed from the *X. arboricola* assemblies using MOB-suite (MOB-recon with default options) [37] implemented within the Galaxy platform.

A pangenomic analysis of the *X. arboricola* genomes was performed. Prior to the analysis, the assembly quality of each genome was assessed using QUAST v5.3.0 [38]. Genome annotation was performed with Bakta v1.12.0 [39], and the resulting GFF3 files were used as input for pangenome reconstruction with Panaroo v1.6.0 [40]. Gene clusters were classified as core genes when present in ≥99% of the genomes. The core-gene alignment generated by Panaroo was used to infer a maximum-likelihood core-genome phylogeny with IQ-TREE2 [41]. The best nucleotide substitution model (GTR+F+I+R10) was selected with ModelFinder [42], and branch support was assessed using 1,000 bootstrap replicates. The final pangenome comprised 12,519 gene clusters, including 2,938 core genes, and the core-genome phylogeny was inferred from an alignment of 3,302,274 bp.

T3SS components and effectors (T3Es), as well as the Xps T2SS components, amylolytic enzymes and CWDE, including 19 cellulolytic, 14 hemicellulolytic and 13 pectinolytic enzymes, were analysed in this study. T3SS, Xps T2SS and CWDE sequences were retrieved from previous works in our group, while T3Es sequences were obtained from the EuroXanth project T3Es database, and the amylolytic enzymes sequences were acquired from the CAZy database [12,43,45]. All these sequences were searched in all the *X. arboricola* proteomes using blastp algorithm [46], and protein hits with a query coverage over 80%, a percentage of identity over 40% and an E-value less than 0.001 were considered as homologues [47]. This uniform identity threshold was applied across protein classes as a conservative criterion to ensure stringency and comparability of results.

Transcription activator-like effectors (TALEs) were predicted and analysed using AnnoTALE [48]. Web blastp verification was performed when necessary. Specific genome region searches were performed using Geneious Mapper (Geneious Prime v2024.0.7). MUSCLE alignment was performed when necessary [49]. SignalP v5.0 was used for signal peptide prediction [50]. InterProScan and NCBI’s Conserved Domain Database (CDD) were used when domain analysis was to be performed [51,52]. It should be noted that some proteins may not be detected in some strains due to potential errors in the annotation of each strain or because some CDSs may be split between two contigs in the case of draft genomes.

### Pepper soft rot test

A soft rot test was conducted on the bacterial strains listed in Table 1 to assess their macerating ability. The strains were selected to represent the phenotypic and genotypic diversity of *X. arboricola,* including the three most relevant pathovars, as well as pathogenic and non-pathogenic strains [53]. Bell peppers were surface sterilized with 70% ethanol for 5 min and 1% sodium hypochlorite for 10 min, rinsed with sterile distilled water three times and dried in a hood. Following this, the bell peppers were cut into fragments of 1.5×1.5 cm and placed on 0.5% water agar plates.

**Table 1.** Strains of *X. arboricola* used in the pepper soft rot test and in the amylolytic activity test

Strain	Host
*X. arboricola* pv. pruni IVIA 2626.1	*P. salicina cv*. Fortune
*X. arboricola* pv. pruni CITA 33	*P. dulcis cv*. Guara
*X. arboricola* pv. juglandis IVIA 1317	*J. regia*
*X. arboricola* pv. juglandis IVIA 2499	*J. regia*
*X. arboricola* pv. corylina CFBP 1846	*C. avellana*
*X. arboricola* pv. corylina IVIA 3978	*C. avellana cv*. Tonda
*X. arboricola* CITA 14	*P. persica*
*X. arboricola* CITA 44	*P. mahaleb*
*X. arboricola* CITA 124	*P. persica*
*X. arboricola* T2.1	*P. avium*
*X. arboricola* T2.2	*P. avium*

Bacterial cultures on LB broth were incubated for 48 h at 27 °C in a rotary shaker, then washed and adjusted to 1×10^8^ c.f.u. ml^−1^ in MgCl_2_ 10 mM and 250 µl of these suspensions were inoculated on bell pepper pieces in triplicate. Mock inoculations were performed by inoculating bell pepper fragments with sterile MgCl_2_ 10 mM. The Petri dishes were closed with Parafilm and incubated at 28 °C for 48 h. Then, the bell pepper pieces were evaluated visually and by probing the slices with a sterile inoculation needle. Bell pepper fragments were considered macerated when they were soft and exhibited water-soaked and dark lesions. The complete experiment was repeated twice.

### Amylolytic activity test

The strains listed in Table 1 were cultured at 27 °C for 48 h in LB broth and then subsequently washed with sterile MgCl_2_ 10 mM. Bacterial suspensions were then adjusted by spectrophotometer to 1×10^8^ c.f.u. ml^−1^, and following this, 5 µl of each suspension was inoculated onto starch agar plates (10 g l^−1^ of starch, 3 g l^−1^ of meat extract and 12 g l^−1^ of agar) in triplicate. After 48 h of incubation, Lugol’s solution (0.2% iodine) was added to the plates. The presence of clear halos around the colonies was interpreted as indicative of positive amylolytic activity. The complete experiment was performed in triplicate.

## Results

### Average Nucleotide Identity (ANI) tree analysis

**Fig. 1.**
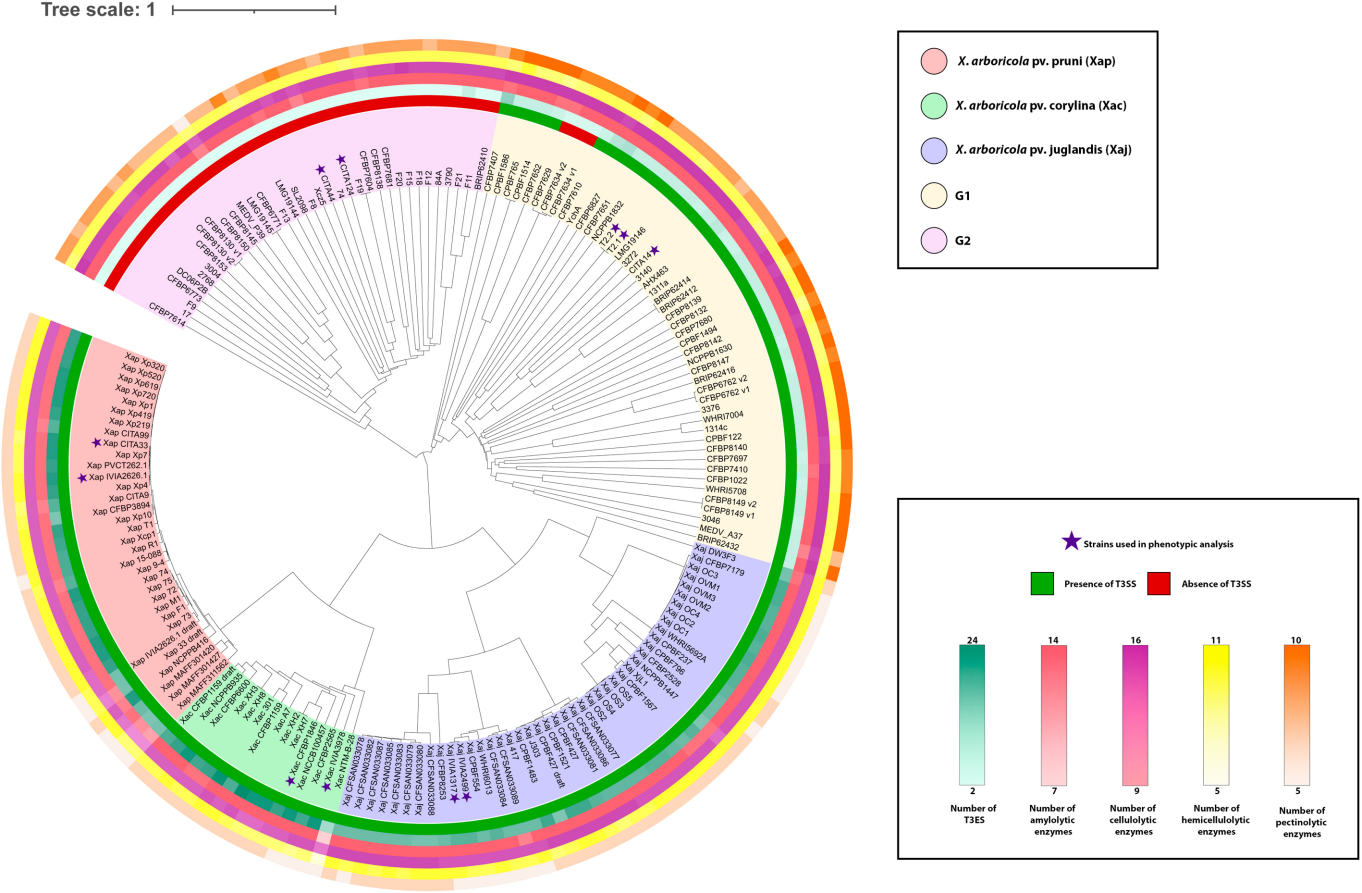
ANI dendrogram, including the genomes of all the *X. arboricola* strains deposited in GenBank by March 2025. Table S3 indicates the presence of the proteins from each of the groups in every strain.

The ANI dendrogram topology exhibited three groups corresponding to each of the main pathovars, *Xap*, *Xaj* and *Xac*. *Xap* strains formed a genomic cluster closer to *Xac* strains (98.68% nucleotide identity) than to *Xaj* strains (97.47%), reflecting higher genetic similarity with the former (Figs 1 and S1; Table S2). Furthermore, *Xaj* strains were subdivided into two groups, with strains presenting 99.34–99.43% ANI within each group and 98.22% between groups. In addition to these clusters, two other groups (G1 and G2) that included strains belonging to the less relevant pathovars and non- or low-pathogenic strains were observed (Table S1). The strains in G1 and G2 exhibited 97.29 and 97.88% average nucleotide identity, respectively, whereas identity between the two groups was only 96.74%. The two strains belonging to pv. celebensis (NCPPB 1630 and NCPPB 1832), both grouped within group G1, but they were not clustered closely among them. Furthermore, strains assigned to pathovar fragariae belonged to both G1 (CFBP 6762 and LMG 19146) and G2 (LMG 19144, CFBP 6773 and LMG 19145=CFBP 6771). Finally, *X. arboricola* pv. arracaciae CFBP 7407 and *X. arboricola* pv. zantedeschiae CFBP 7410 were classified within G1.

**Fig. 2.**
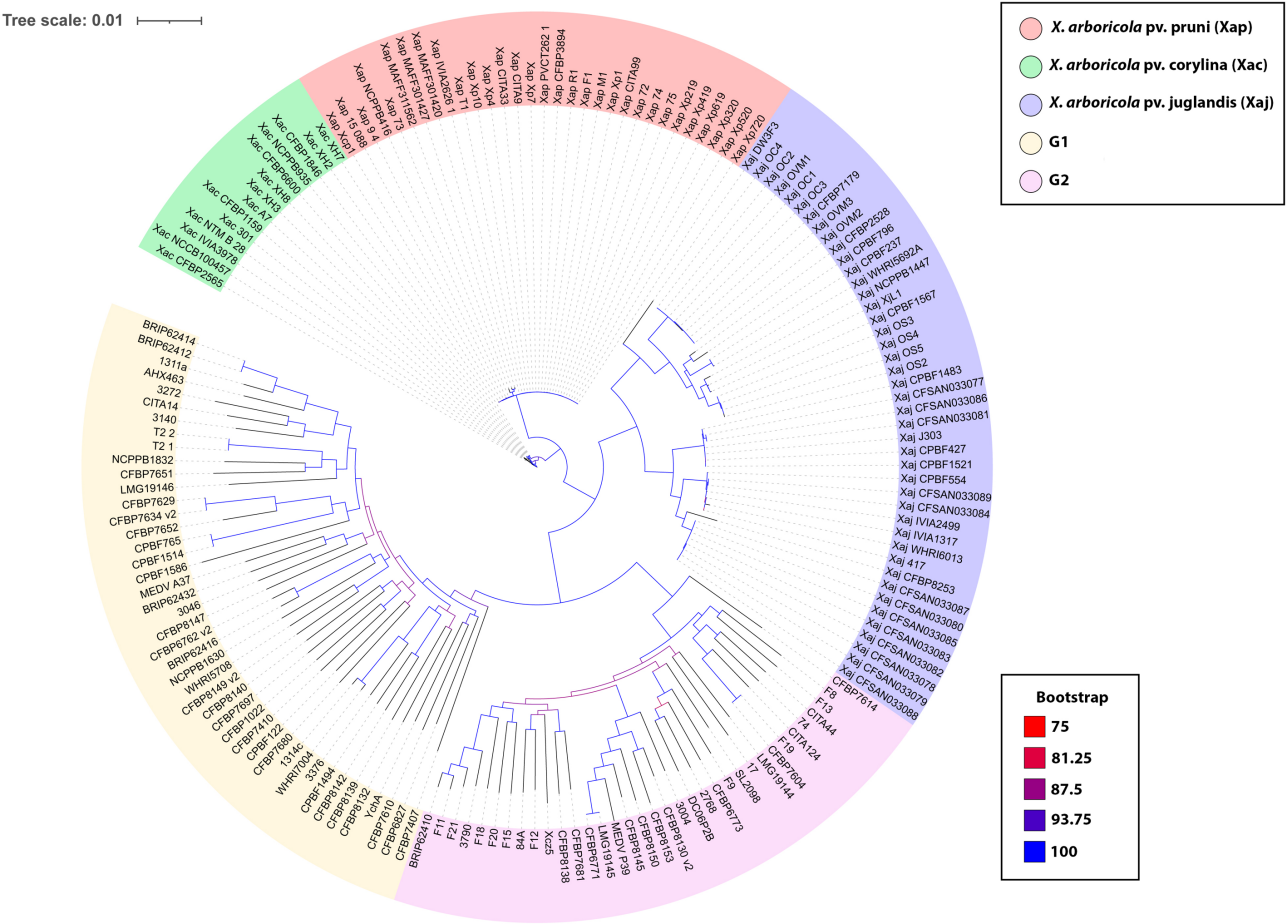
Phylogenetic tree based on the core genome of *X. arboricola*.

The phylogenetic tree based on the core genome also presented a similar clustering pattern. *Xap*, *Xaj* and *Xac* each formed separate clades, with the *Xaj* clade being further divided into two subclades (Fig. 2). Additionally, two additional clades were observed, corresponding to strains in groups G1 and G2 according to the ANI dendrogram.

### Plasmid content

Among the *X. arboricola* strains deposited in the NCBI, only those with complete genomes harboured plasmid sequences deposited, which hindered the overall analysis of *X. arboricola* plasmid content. The use of MOB-recon allowed the reconstruction of some plasmids from the *X. arboricola* assemblies, regardless of their assembly level. Homologues to pXap41 (FR875157) were found in all the *Xap* genomes, except for the strain 15-088. However, plasmid content in *Xaj* and *Xac* strains was scarce. Strains in G1 harboured no plasmids, while in G2, only strain F21 harboured a plasmid (Table S1).

### Comparative genomics of type III secretion system (T3SS) and type III effectors (T3Es) in *X. arboricola*

T3SS components and regulators were searched in all the *X. arboricola* proteomes (Table S3). Strains that presented five or fewer T3SS-related proteins were considered T3SS-negative, while the remaining strains showed from 21 to 28 T3SS-related proteins and were considered T3SS-positive. T3SS-positive strains comprised pathovars *Xap*, *Xaj* and *Xac*, as well as strains in G1, except for strains CFBP 7629 and CFBP 7634, which lacked this secretion system. Moreover, all the strains in G2 lacked the T3SS (Fig. 1, Table 2).

Different numbers of T3Es were found in the *X. arboricola* strains (Fig. 1; Table S3). Strains belonging to pathovars *Xap*, *Xaj* and *Xac* presented from 13 to 24 T3Es, except for NTM-B-28, which harboured only nine. The strain NTM-B-28 fails the completeness check and presents many frameshifted proteins according to NCBI Genome notes, and consequently, the absence of some components would not be considered as true, instead caused by completeness issues. Strains belonging to G1 harboured three to eight, except for *X. arboricola* pv. arracaciae CFBP 7407, which presented 11 T3Es. Strains in G2 harboured two T3Es, XopAW and XopAZ, except for strains 17 and F21, which showed one and three additional T3Es, respectively. Moreover, only six putative TALEs were identified across all the species, exclusively in strains *Xac* A7, CFBP 1159, IVIA 3978, NCPPB 935, XH8 and *Xap* 9-4.

### Comparative genomics of Xps T2SS components and cell wall-degrading enzymes (CWDE) in *X. arboricola*

The 11 components of the Xps T2SS were found in all *X. arboricola*, except for four strains (Table S3). MAFF 301420 and the draft genome of IVIA 2626.1 lacked one of the components. However, a detailed observation of their annotation revealed that these genes would have been truncated between two contigs. NTM-B-28 presented three frameshifted components of the Xps T2SS, *xpsI*, *xpsM* and *xpsN*, which could be due to the completeness problems of this genome. Finally, MEDV_A37 had an internal stop in *xpsG*.

Regarding the CWDE, the content of cellulolytic, hemicellulolytic and pectinolytic enzymes of *X. arboricola* strains was assessed. In general, the strains with higher cellulolytic enzymes content belonged to G1 and G2, presenting from 13 to 16 of these enzymes. Moreover, all the *Xaj* strains harboured 14 or 15 cellulolytic enzymes, except for NCPPB 1447, which contained only 12. *Xac* strains harboured from 11 to 14 of these enzymes, while *Xap* strains possessed from 9 to 13 (Fig. 1; Table S3).

Furthermore, *Xap* and *Xaj* strains harboured from 9 to 11 hemicellulolytic enzymes, while *Xac* presented from 8 to 11, except for strain NTM-B-28, which harboured only five. Strains in G1 and G2 possessed from eight to ten, except for strain CPBF 1586 in G1, which harboured 11 (Fig. 1; Tables 2 and S3).

**Table 2.** Summary of repertoire of T3SS, T3Es, cell-wall degrading and amylolytic enzymes present in all the *X. arboricola* strains deposited in GenBank by March 2025

	T3SS	T3E	Cellulolytic enzyme	Hemicellulolytic enzyme	Pectinolytic enzyme	Amylolytic enzyme
***Xap***	+	**19** (13 to 21)	**13** (9 to 13)	**11** (9 to 11)	**6** (5 to 6)	**13** (10 to 13)
***Xac***	+	**21** (18 to 24*)	**13** (11 to 14)	**10** (8 to 11*)	**6** (5 to 6)	**14** (13 to 14*)
***Xaj***	+	**16** (13 to 19)	**14** (12 to 15)	**11** (9 to 11)	**6** (5 to 6)	**14** (12 to 14)
**G1**	+†	**5** (3 to 11)	**16** (15 to 16)	**10** (8 to 11)	**9** (7 to 10)	**14** (12 to 14)
**G2**	Negative	**2** (2 to 5)	**15** (13 to 16)	**10** (9 to 10)	**8** (7 to 9‡)	**14** (12 to 14)

Bold numbers represent average counts, while values in parentheses indicate the minimum–maximum range. Average counts exclude NTM-B-28.

*The strain NTM-B-28, which fails completeness check and presents many frameshifted proteins according to NCBI Genome notes, exhibits lower counts.

†Except for CFBP 7629 and CFBP 7634.

‡MEDV_P39 (scaffold genome) only harboured five pectinolytic enzymes.

Regarding the pectinolytic enzymes, the three main pathovars harboured five or six enzymes, whereas most of the strains in G1 and G2 exhibited seven to ten. The only exception was strain MEDV_P39, which harboured only five of these enzymes (Fig. 1; Tables 2 and S3).

### Pepper soft rot test

**Fig. 3.**
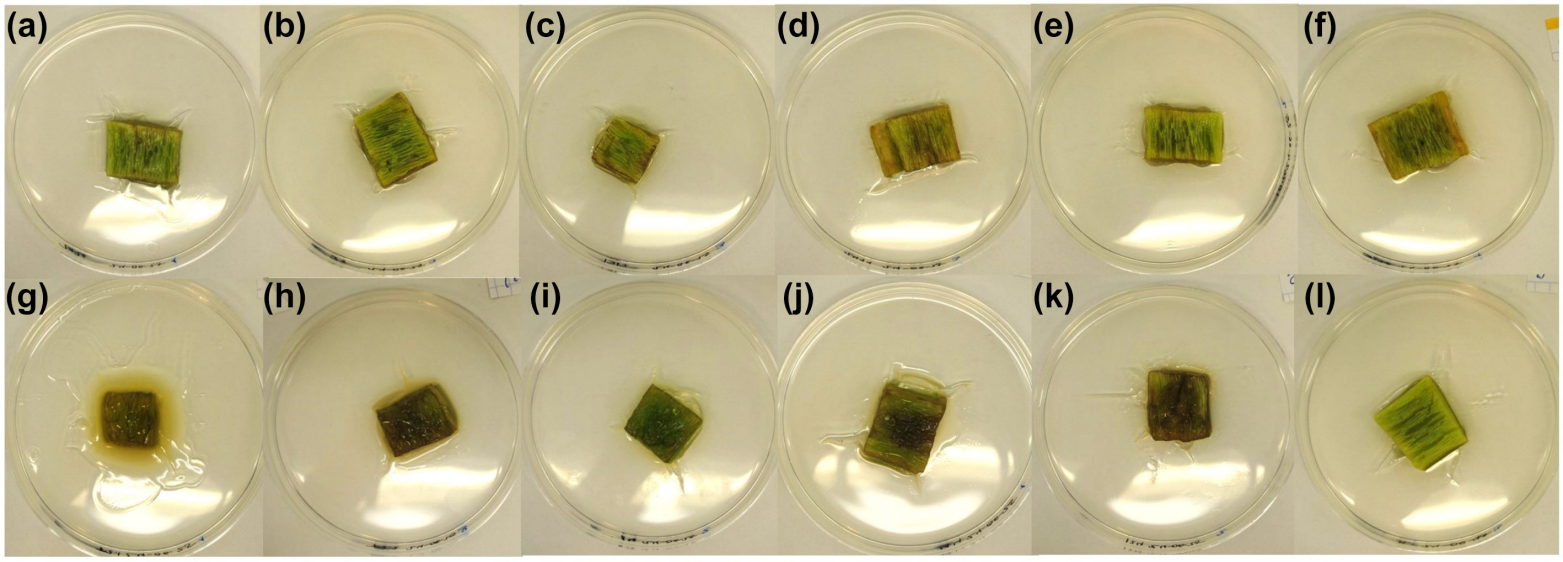
Evaluation of soft rot symptoms produced by *X. arboricola* strains on bell pepper fruits at 48 h post-inoculation. (a) IVIA 2626.1 (*X. arboricola* pv. pruni). (b) CITA 33 (*X. arboricola* pv. pruni). (c) IVIA 1317 (*X. arboricola* pv. juglandis). (d) IVIA 2499 (*X. arboricola* pv. juglandis). (e) CFBP 1846 (*X. arboricola* pv. corylina). (f) IVIA 3978 (*X. arboricola* pv. corylina). (g) CITA 14 (*X. arboricola*, G1). (h) CITA 44 (*X. arboricola*, G2). (i) CITA 124 (*X. arboricola*, G2). (j) T2.1 (*X. arboricola*, G1). (k) T2.2 (*X. arboricola*, G1). (l) Mock.

Soft rot test performed on bell pepper showed that strains from G1 and G2, CITA 14, CITA 44, CITA 124, T2.1 and T2.2, produced dark, water-soaked lesions and softening of tissues, indicating that these strains were able to macerate the pepper. On the contrary, IVIA 2626.1, CITA 33, IVIA 1317, IVIA 2499, CFBP 1846 and IVIA 3978, belonging to *Xap*, *Xaj* and *Xac*, respectively, remained firm and dry, exhibiting only some dark spots (Fig. 3).

### Analysis of the amylolytic activity and comparative genomics of amylolytic enzymes

**Fig. 4.**
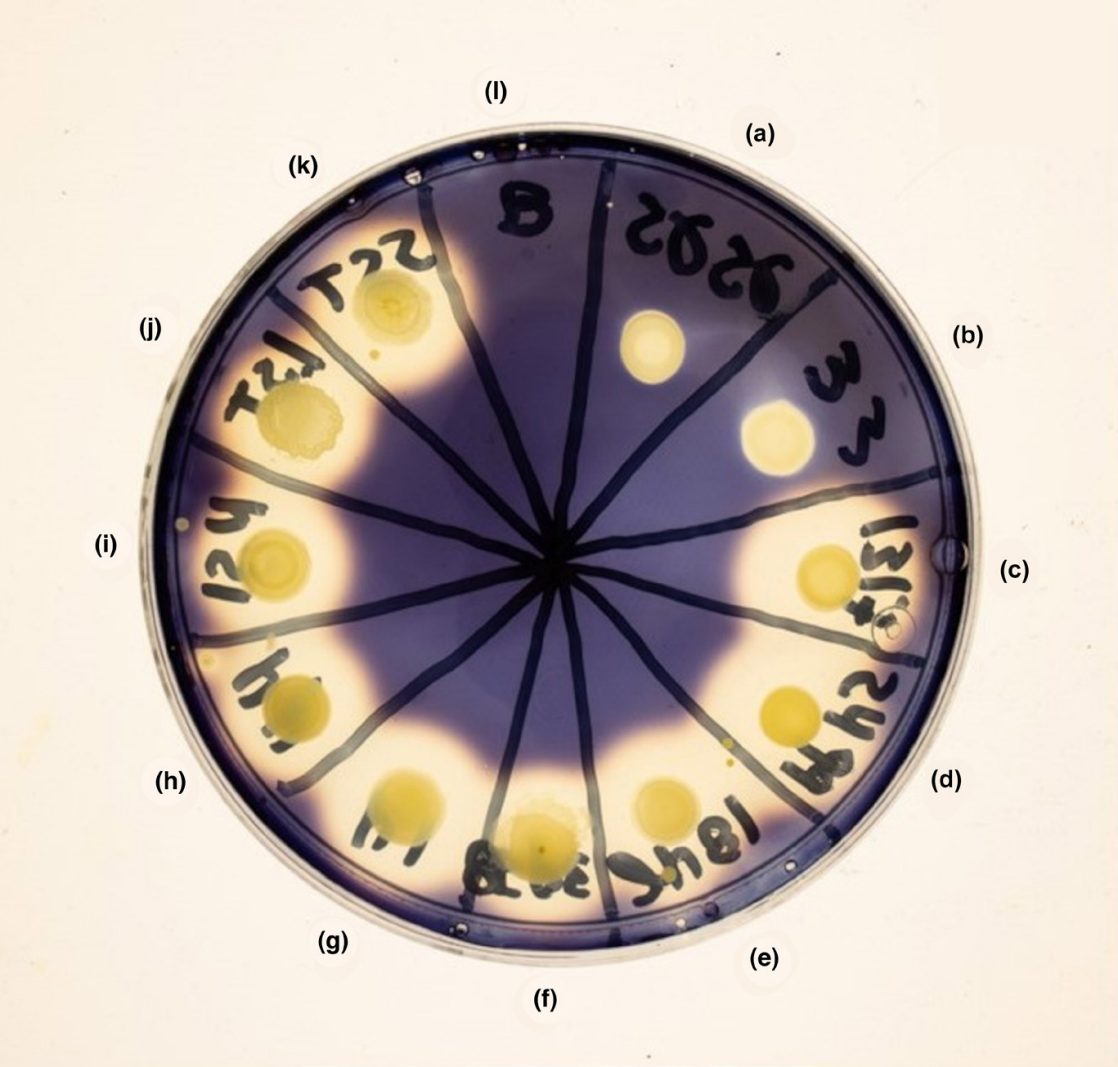
Assessment of starch hydrolysis by *X. arboricola* after 48 h of incubation. (a) IVIA 2626.1 (*X. arboricola* pv. pruni). (b) CITA 33 (*X. arboricola* pv. pruni). (c) IVIA 1317 (*X. arboricola* pv. juglandis). (d) IVIA 2499 (*X. arboricola* pv. juglandis). (e) CFBP 1846 (*X. arboricola* pv. corylina). (f) IVIA 3978 (*X. arboricola* pv. corylina). (g) CITA 14 (*X. arboricola*, G1). (h) CITA 44 (*X. arboricola*, G2). (i) CITA 124 (*X. arboricola*, G2). (j) T2.1 (*X. arboricola*, G1). (k) T.2.2 (*X. arboricola*, G1). (l) Mock.

Phenotypic assays for amylolytic activity on starch agar plates showed that *Xaj* strains IVIA 1317 and IVIA 2499, *Xac* strains CFBP 1846 and IVIA 3978, as well as strains not assigned to any pathovar (CITA 14, CITA 44, CITA 124, T2.1 and T2.2), were able to hydrolyse starch, as indicated by the formation of clear halos. In contrast, *Xap* strains, IVIA 2626.1 and CITA 33, did not produce halos, indicating the absence of amylolytic activity (Fig. 4).

Furthermore, 14 amylolytic enzymes were searched in the *X. arboricola* strains by blastp. *Xaj*, G1 and G2 strains harboured from 12 to 14 amylolytic enzymes, while *Xac* presented 13 or 14, except for strain NTM-B-28, which harboured only seven. *Xap* strains harboured from 10 to 13 of these enzymes (Fig. 1; Table S3).

## Discussion

*X. arboricola* species is characterized by a high degree of genetic and ecological variability, encompassing strains that differ markedly in host range, pathogenicity and economic relevance. Within this context, both ANI and phylogenetic analyses showed the same topology, corresponding to a structured pattern in which those belonging to the pathovars *Xap*, *Xaj* and *Xac* were grouped into well-defined, distinct clusters. Moreover, these three pathovars were separated from strains belonging to less economically relevant pathovars, as well as from saprophytic, opportunistic and weakly or non-pathogenic strains (belonging to G1 and G2) [5,11, 12, 27, 54]. These results are consistent with the analysis performed by Cesbron *et al*. [27] and this agreement is substantially reinforced here by phylogenetic analysis and the inclusion of a broader and ecologically more representative set of strains, particularly through the incorporation of isolates from a wide range of hosts, thereby expanding the ecological context and enhancing the robustness of the study. It is also in accordance with previous phylogenetic analysis based on MLSA of four (*dnaK*, *fyuA*, *gyrB* and *rpoD*) or seven (*atpD*, *dnaK*, *efp*, *fyuA*, *glnA*, *gyrB* and *rpoD*) housekeeping genes or on the core genome of the species, all of which were conducted using a limited number of strains [5,11, 12, 54].

Moreover, in the present ANI dendrogram, *Xaj* strains clustered into two distinct subgroups; however, no clear explanation for this divergence could be identified based on the available data on disease symptomatology, strain origin, or other relevant factors (Table S1). Furthermore, this clustering was supported by the phylogenetic analysis.

Concerning the less relevant pathovars, the divergence between G1 and G2 could not be conclusively linked to geographical origin or classification of the host of isolation (e.g. monocotyledonous or dicotyledonous plants) (Table S1). Moreover, it is important to note that the two strains belonging to pv. celebensis were grouped in G1 but separated from each other, whereas the strains of pv. fragariae were dispersed throughout the dendrogram. These data indicate that, in contrast to the observations for pathovars *Xap*, *Xaj* and *Xac*, where genomic evidence supports their classification, such differentiation is not observed in pathovars celebensis and fragariae. This lack of clustering among strains of the same pathovar is also consistent with our phylogenetic tree based on the core genome of the species, as well as with phylogenies based on MLSA [5,54]. Average Amino Acid Identity analysis by Gétaz *et al*. showed that *X. arboricola* pv. fragariae isolates clustered in two groups, with strains CFBP 6773, LMG 19144 and LMG 19145 (syn. CFBP 6771) separated from CFBP 6762 and LMG 19146 [55]. This pattern matches the clustering observed in the present ANI analysis. It should be noted that, as *X. arboricola* pv. arracaciae CFBP 7407 and *X. arboricola* pv. zantedeschiae CFBP 7410 were the only strains deposited in GenBank for these pathovars, therefore, no definitive conclusions could be drawn regarding their position in the ANI dendrogram.

Other strains isolated from the same host were also scattered through G1 and G2, not clustering together, regardless of their symptomatology similarities. This is the case of the tomato strains BRIP62410, BRIP62412, BRIP62414, BRIP62416 and BRIP62432 from Australia, which clustered into both G1 and G2; and for CPBF 1494 and CPBF 765 isolated from *Carya illinoinensis* in Portugal, which clustered within G1 at separate positions; importantly, these latter two strains are misclassified in GenBank as Xaj [56,57]. Furthermore, several strains belonging to G1 and G2 have been described to exhibit doubtful pathogenicity or have remained unreported since their initial isolation [11,13,55, 57].

All the *Xap* strains except for 15-088 harboured the plasmid pXap41, which was absent in the genomes belonging to other pathovars or with no pathovar affiliation. This result confirmed the observations of Pothier *et al*. and Garita-Cambronero *et al.,* whose analyses were based on a more limited number of genomes [11,58].

Concerning the T3SS, genomic analysis confirmed its presence in pathovars *Xap*, *Xaj* and *Xa*c and in all G1 strains, except for CFBP 7634 and CFBP 7629, while none of the strains in G2 harboured it. The absence of T3SS in strain CFBP 7634 has previously been reported by Garita-Cambronero *et al*. and Cesbron *et al*. [12,27]. Cesbron *et al*. demonstrated that in T3SS-positive strains, genes encoding the T3SS are flanked by *ltaE* and *trpG*. In contrast, in CFBP 7634, these genes delimited a genomic region containing genes encoding a Type 1 Restriction-Modification System, which is known to be involved in phage defence in *Escherichia coli* [27]. To confirm whether this group of genes was present in all the T3SS-negative strains, the presence of the Type 1 Restriction-Modification System region was investigated across the strain collection used in this work. While the region bordered by the *ltaE* and *trpG* genes was clearly identified in strain CFBP 7629, it was absent in G2 strains. Instead, these strains showed a distinct genomic organization upstream of *trpG*, predominantly comprising *ltaE* and hypothetical protein-encoding genes, highlighting variability in the genomic context associated with T3SS absence.

Moreover, T3SS-negative strains carried homologues of HrpX, HrpG, HpaS, HpaR2 and HrcN, except CFBP 7614 and CITA 124, which lacked HrpX and HrcN, respectively. HrpG and HrpX are regulators that control the expression of T3SS components and some T3Es, and also regulate T2SS substrates, chemotaxis and flagellar genes [59]. Furthermore, previous studies in *Xanthomonas campestris* pv. campestris have shown that HpaS would form a two-component regulatory system along with HrpG, while HpaR2 would form another two-component regulatory system with HpaS [60]. Although these regulatory interactions have not been experimentally validated in *X. arboricola*, the presence and conservation of the homologous proteins (HpaS, HrpG and HpaR2) suggest that similar regulatory mechanisms may operate in this species. Finally, the HrcN reference sequence exhibited ~45–50% identity to their putative homologues in these T3SS-negative strains. In addition, when these putative homologues were searched against all non-redundant protein sequences in GenBank, they showed a high query coverage and identity against the flagellar component FliI (100% Query Coverage and ~100% Identity). This would imply that the putative homologues to HrcN found in these strains were actually homologues to FliI. The aforementioned would explain the presence of T3SS-related proteins in putative T3SS-negative strains, as the proteins identified in these strains correspond either to regulators involved in processes other than T3SS export or, in fact, to proteins unrelated to T3SS. T3SS is thought to be fundamental for virulence in pathogenic *Xanthomonas* and has been found missing in non-pathogenic and opportunistic bacteria [61,62]. Regarding the less economically relevant pathovars, the presence or absence of this secretion system is associated with each strain membership in G1 or G2. In this way, *X. arboricola* pv. arracaciae, pv. celebensis and pv. zantedeschiae strains belonged to G1 and harboured a T3SS, whereas pv. fragariae strains were split between G1 with T3SS and G2 without T3SS, and this is consistent with previous studies [54,55, 62].

Moreover, diverse T3Es profiles were found among strains. The highest amount of T3Es was observed in the strains belonging to the three main pathovars, which confirms previous observations in the species [12,27, 63]. *Xac* carried an average of 21 T3Es, followed closely by *Xap* strains with 19 T3Es, while *Xaj* displayed the most limited repertoire with 16 T3Es. Furthermore, strains belonging to G1 presented an average of five effectors, while the lowest number of T3Es was observed in G2, which harboured an average of two [12,27, 63]. Among the strains in G1, the only two T3SS-negative ones, CFBP 7634 and CFBP 7629, exhibited the smallest T3Es repertoire within this group, harbouring three T3Es. Cesbron *et al*. also observed a small T3Es repertoire in CFBP 7634 and hypothesized that, as this strain lacked HrpF, those T3Es would not be translocated into the plant cell [27]. This hypothesis could also be applied to the strains clustered in G2.

Regarding the strains of the less economically relevant pathovars, it is also noteworthy that *X. arboricola* pv. celebensis NCPPB 1630 and NCPPB 1832 and *X. arboricola* pv. fragariae LMG 19146 and CFBP 6762, which belonged to G1, harboured the most frequent set of T3Es found in G1. This set comprised AvrBs2, XopF1, XopM, XopAW and XopAZ. However, *X. arboricola* pv. fragariae LMG 19144, LMG 19145 and CFBP 6773, which belonged to G2, only harboured XopAW and XopAZ, which was the most frequent set of effectors in G2. This data indicates that, for pathovar fragariae, the repertoire of T3Es in a strain is more closely associated with its classification into G1 or G2 than with its classification as part of the fragariae pathovar itself. On the other hand, *X. arboricola* pv. zantedeschiae CFBP 7410 contained AvrBs2, XopF1, XopAW and XopAZ, as in other strains from G1, but also carried XopAU, which was unique for this strain within *X. arboricola* species. Moreover, *X. arboricola* pv. arracaciae CFBP 7407 harboured two unique effectors, XopAH and XopAG1, which were absent from the remaining *X. arboricola* strains.

Furthermore, while some authors had previously described differences in T3Es repertoires within a single pathovar depending on the disease produced (e.g. WBB or VOC, in the case of *Xaj* strains) or on the host (e.g. *C. avellana* or *Corylus maxima*, for *Xac* strains), such differences were not observed in this study. Cesbron *et al*. described XopAI and XopB as unique for the VOC-causing strain CFBP 7179 [27]. Additionally, XopB was described by Hajri *et al*. as unique for VOC-causing strains [63]. Nevertheless, in the present analysis, XopB was detected not only in CFBP 7179 but also in *X. arboricola* pv. arracaciae CFBP 7407 and in other *Xaj* strains lacking disease descriptions in GenBank (OC1, OC2, OC3, OC4, OVM1, OVM2 and OVM3), whereas XopAI was also observed in some WBB-causing strains (Tables S1 and S3). Moreover, it is important to highlight that the limited number of some disease- and host-associated strains available in GenBank likely precludes robust conclusions regarding the association between single effectors and symptom specificity or host range. However, it is evident that in most cases, pathogen-host interactions and disease development are determined by a combination of virulence factors rather than isolated ones. Nevertheless, one T3E, XopE3, exhibited a clear specific association with *Xap*, being detected in all its strains except Xp10 and Xp4, in which the sequence appeared to be split across two contigs, resulting in an apparent truncation. This ubiquity within the pathovar would suggest that this effector could play an important role in pathogenesis. Furthermore, the gene *xopE3* has been previously described as a unique effector of *Xap* within *X. arboricola*, in studies involving smaller bacterial collections and has also been used as a PCR target for *Xap* detection [11,12, 63].

Some T3Es were widely distributed in *X. arboricola*. For instance, AvrBs2 was present in the core T3Es repertoire of *Xac* and G1, as well as in the majority of the strains from *Xap* and *Xaj*. Additionally, XopAV2 was found in the core of the three pathovars and in none of the strains from G1 and G2. XopAW and XopAZ were found in all *X. arboricola* strains, except for NCPPB 1447, which lacked XopAW. Furthermore, AvrXccA2, XopL, XopQ and XopZ1 were present in the core T3Es repertoires of *Xap* and *Xaj* strains, as well as in all the *Xac* strains except for NTM-B-28, and were absent in G1 and G2. XopK was found in the core T3Es repertoires of *Xap* and *Xaj*, in strain 17 from G2 and in all the *Xac* strains except for NTM-B-28. Moreover, XopN was also found in the core of *Xap* and *Xaj*, in CFBP 7407 and in all *Xac* strains except for NTM-B-28 (Table S3). The presence of a higher amount of T3Es in *X. arboricola* pv. arracaciae CFBP 7407 with respect to other strains in G1, as well as the presence of two unique effectors in this strain, XopAH and XopAG1 and some T3Es that are not present in other G1 strains, such as XopN or XopB, may be related to its host, *A. xanthorrhiza*. However, more genomes from this pathovar would be needed for confirmation.

Only six putative TALEs were detected in *X. arboricola* supporting the limited presence of these sequences within the species [63,65]. Five of them were found in pv. corylina (strains A7, CFBP 1159, IVIA 3978, NCPPB 935 and XH8), while only one was found in a *Xap* strain (*Xap* 9-4), encoded within a plasmid. Among them, only the TALEs in strains A7 and NCPPB 935 could be functional, as they were complete proteins that contained a minimum of 6.5 repeats, which are required for the induction of target gene expression [63,66].

The Xps T2SS, responsible for the secretion of some CWDE [16,67], was identified in all strains, excluding four exceptions (NTM-B-28, first draft of IVIA 2626.1, MAFF 301420, MEDV_A37) in which some components were absent or appeared frameshifted. The absence of some components of the system in NTM-B-28 could be due to the completeness issues of this genome. In IVIA 2626.1 and MAFF 301420, these absences were likely due to draft genome fragmentation rather than true gene loss, as both correspond to draft genomes sequenced using IonTorrent technology. This was supported by the complete genome of IVIA 2626.1, in which all the components were identified [68]. In contrast, the absence of *xpsG* in MEDV_A37 genome appeared to be real and likely not due to annotation, assembly or other artefacts, and may impact CWDE secretion. In *Xanthomonas oryzae* pv. oryzae, disruption of Xps T2SS components impaired the secretion of cellulases and xylanases [67,69], while in *X. campestris* pv. vesicatoria Xcs T2SS components partially complemented the absence of their homologous Xps counterparts [70]. Thus, the blastp search of the Xcs components in strain MEDV_A37 was performed, revealing that this strain harboured a complete Xcs T2SS.

Strains belonging to G1 exhibited the highest number of cellulolytic enzymes, with an average of 16, followed by those in G2 and *Xaj*, which harboured averages of 15 and 14 enzymes, respectively. In contrast, *Xac* and *Xap* contained an average of only 13 cellulolytic enzymes. The majority of cellulolytic enzymes analysed were widely distributed across all the strains, with AAM40701.1 and AAM39347.1 being present in all *X. arboricola*. However, some others were more group-specific, as is the case of cellulase AAM38359.1, which was present in all the *Xaj*, G1 and G2 strains, but absent in all strains belonging to pvs *Xap* and *Xac*. This supports the observations of Garita-Cambronero *et al*. regarding this enzyme [11] while extending them through the analysis of a larger and more diverse strain set, thereby strengthening the reliability of the conclusions.

Cellulolytic enzymes have been reported to contribute to the virulence of several *Xanthomonas* species. For instance, the endoglucanase BglC3 has been identified as necessary for full virulence in *Xanthomonas citri* subsp. citri [71]. However, the relationship between these enzymes and virulence remains inconsistent. While some studies report that mutations in cellulolytic enzyme genes reduced virulence, others have shown only minor or no effects [71,74]. For example, Yun *et al*. demonstrated that cellulase production by *X. oryzae* pv. oryzae did not play a key role in rice pathogenesis, suggesting a potential function in saprophytic growth on dead plant tissues instead [72]. Additionally, certain cellulolytic enzymes have been proposed to be involved in bacterial progression during the colonization stage [71].

Regarding the hemicellulolytic enzymes, *Xap* and *Xaj* strains harboured, on average, 11 enzymes, whereas strains in G1 and G2 and *Xac* presented an average of ten. Notably, AAM41046.1 and AAM39463.1 were conserved across all the *X. arboricola* strains, whereas AAM43326.1 and AAM39089.1 were consistently detected in all the strains except for NTM-B-28. Moreover, AAM41676.1 was only found in *Xap*, *Xaj* and *Xac*, but not in G1 or G2 strains. Garita-Cambronero *et al*. observed the presence of the xylosidase/arabinosidase NP_637752.1, equivalent to AAM41676.1, in strains belonging to the three main pathovars but not in non-or low-pathogenic strains or in those belonging to pv. celebensis, which corresponds to the observations in the present study. However, they reported the presence of the xylanase NP_638385.1, equivalent to AAM42309.1, in several *Xap* strains and in others not associated with *Prunus*, while in the present study this enzyme was only observed in CPBF 1586 [11]. Moreover, some hemicellulolytic enzymes may affect virulence. Rajeshwari *et al*. observed that mutations in the *xynB* gene from *X. oryzae* pv. oryzae, which codes for a secreted xylanase, partially affected virulence [75]. Furthermore, Solé *et al*. showed that the xylanases XCV4358 and XCV4360 from *X. campestris* pv. vesicatoria contributed to virulence and bacterial multiplication in planta [19].

Major differences were observed in pectinolytic enzyme content, with higher values in strains belonging to G1 and G2. While the three pathovars harboured an average of six pectinolytic enzymes, the strains in G1 and G2 presented an average of nine and eight enzymes respectively. This pattern is consistent with the results of the soft rot test, in which strains CITA 14, CITA 44, CITA 124, T2.1 and T2.2 (G1 and G2) were able to macerate pepper tissues, whereas strains IVIA 2626.1, CITA 33, IVIA 1317, IVIA 2499, CFBP 1846, IVIA 3978, belonging to pvs. *Xap*, *Xaj* and *Xac* showed no maceration ability. Previous research also supports this observation of low-pathogenic *X. arboricola* strains being positive to the soft rot test. Sawada *et al*. described that genetically diverse *X. arboricola* strains isolated from bacterial spot disease of grapevine were weak, opportunistic pathogens and were able to cause soft rot in potato [76,77]; in contrast to *Xap*, which could not macerate potatoes. Furthermore, Vandroemme *et al*. reported that *X. arboricola* pv. fragariae induces potato maceration, suggesting a saprophytic rather than a pathogenic lifestyle [62].

Concerning individual pectinolytic enzymes, some of them were widely distributed in the species, such as AAM42729.1, which was found in all the *X. arboricola* strains except in F9. Other enzymes seem to be more specific for certain groups, and for instance, the pectate lyase E (AAM39441.1) was present in all the G1 and G2 strains and absent in *Xap*, *Xaj* and *Xac*. Functional analysis should be performed to determine whether this enzyme is related to the differential maceration capacities observed in the soft rot experiments. Moreover, other pectinolytic enzymes, such as AAM39440.1 and AAM41544.1, were predominantly present in G1 and G2 strains and were absent in the three main pathovars. In contrast, a degenerated pectate lyase, AAM37225.1, was found in most of the strains from pvs. *Xap*, *Xaj* and *Xac*, but was absent in G1 and G2 strains. Several studies indicate that pectinolytic enzymes play a limited role in *Xanthomonas* virulence. For instance, they appear to have only a minor effect in *X. oryzae* pv. oryzae, while in *X. campestris* pv. campestris, the polygalacturonase PehA contributes modestly to virulence in cabbage and PghAxc and PghBxc are required for full virulence in *Arabidopsis* [78,81]. Furthermore, Tayi *et al*. proposed that the ability to macerate potato tuber slices and pepper fruits of some *X. campestris* pathovars, which do not cause soft rot diseases in growing plants, could indicate that *Xanthomonas* may, at some time in their life cycle, macerate some plant tissues on their host or on a non-host, which would justify the maintenance and expression of pectinolytic genes in their genomes [80] and would also support the observations in the present study regarding *X. arboricola*.

Starch, which consists of two polysaccharides, amylose and amylopectin, is a major reserve substance in plants and is found in fruits, seeds, roots, leaves and stems [20]. Starch hydrolysis is catalysed by several amylolytic enzymes belonging to glycoside hydrolases (GH): α-amylases, of which the main CAZy group is GH13; β-amylases, which are embodied by GH14; and glucoamylases, which correspond to GH15 [82]. In addition, some authors suggest that families GH57, GH119 and GH126 would also be considered α-amylase families [83]. However, none of these, nor GH14, are found in *Xanthomonas* according to CAZy database [78]. Nevertheless, *Xaj* contains GH15 and, notably, GH13, exhibiting a higher number of enzymes from this last family than other members of the *Xanthomonas* genus [78]. The present study expands the knowledge about the amylolytic enzyme content of *X. arboricola* and their activity, focusing on intraspecies differences.

In the present study, *Xap* strains, IVIA 2626.1 and CITA 33, were not able to hydrolyse starch, while the rest of the strains, belonging to pvs. juglandis and corylina and clusters G1 and G2 did hydrolyse this polysaccharide. This supports previous observations in the species that demonstrated that *Xap* strains, such as Xp1 or Xp10, did not hydrolyse starch, whereas strains belonging to *Xaj*, *Xac*, G1 and G2 did hydrolyse it [2,4,9, 57, 84]. To assess genomic differences that could explain this condition, the amylolytic enzymes of all the *X. arboricola* strains were analysed. A plausible explanation lies in the differences in enzyme content: *Xaj*, *Xac* and G1 and G2 strains harboured an average of 14 amylolytic enzymes, whereas *Xap* strains contained an average of only 13. Upon analysing the individual amylolytic enzymes, it was evident that nearly all were found in almost all the strains, except for AKU48857.1, which was absent in *Xap* strains but present in the core of G1 and G2 and most *Xaj* and *Xac* strains. When specifically considering the strains used in the assay, it was observed that AKU48857.1 was present in IVIA 1317, IVIA 2499, CFBP 1846, IVIA 3978, CITA 44, CITA 14, CITA 124, T2.1 and T2.2, while it was absent in IVIA 2626.1 and CITA 33. Nonetheless, the contribution of AKU48857.1 to starch utilization is unlikely to occur at the initial stage, but rather in the processing of secondary products, as this enzyme belongs to the glycoside hydrolase family 13, subfamily 23 (GH13_23). This subfamily includes enzymes with oligo exo-α-1,6-glucosidase, α-glucosidase, trehalose α-1,1-glucosidase and α-glucosyltransferase activities, but not α-amylase [89]. Homologues to AKU48857.1 are predicted to hydrolyse α (1→6)-d-glucosidic linkages in isomaltose and some oligosaccharides produced from starch by α-amylase, as well as the terminal, non-reducing (1→4)-linked α-d-glucose residues. Therefore, these enzymes would likely act synergistically with α-amylase in starch hydrolysis, but play a secondary role. Moreover, SignalP analysis showed that AKU48857.1 lacked both Sec and Tat signal, suggesting that it is either secreted via OMV, as reported for other enzymes [19], or localized in the cytoplasm and, therefore, would not directly contribute to the extracellular starch metabolism.

To assess if other differences between amylolytic enzymes could be responsible for phenotypic results, the putative extracellular amylolytic enzymes containing signal peptides were analysed. SignalP analysis showed that AKU48853.1 and AKU52271.1 contained Sec signal peptides, whereas the remaining enzymes lacked Sec or Tat signals. Among them, only AKU52271.1 harboured α-amylase molecular function determined with InterProScan, while, according to CDD, AKU48853.1 harboured a maltogenic amylase C-terminal domain (Table S4A). Analysis of AKU52271.1 and AKU48853.1 homologues across *X. arboricola* strains revealed that certain mutations, including amino acid insertions, deletions and substitutions, were exclusively found in *Xap* strains (Table S4B, C). Subedi *et al*. observed that even single-base mutations led to the loss of the amylolytic activity of α-amylase and to the inability to elicit the HR in pepper plants containing the Bs2 resistance gene by AvrBs2 from *X. euvesicatoria* [90]. Whether the mutations in AKU52271.1 and AKU48853.1 homologues would affect the amylolytic activity remains unknown, as well as if some of the amylolytic enzymes would play any role in pathogenesis [23]. Further research, including functional analysis, is needed to clarify the role of amylolytic enzymes in *X. arboricola*, the impact of its mutations, and whether other factors, like regulatory proteins or the Rpf/DSF quorum-sensing system would contribute to the differences in starch hydrolysis between *Xap* and other *X. arboricola* [23].

This study revealed that ANI analysis grouped *X. arboricola* strains according to major pathovars (*Xap*, *Xaj* and *Xac*), while other strains clustered into G1 and G2, comprising low- or non-pathogenic, saprophytic and opportunistic isolates [11,13,55, 57]. These groups also differed in virulence-related features, with reduced T3SS and T3E repertoires and potential variation in CWDE and amylolytic enzyme involvement [11,13,55, 57]. Comparative analysis of hydrolytic enzyme profiles revealed distinct patterns among the studied strains. Although *Xap* and *Xaj* strains contained higher numbers of hemicellulolytic enzymes, the three pathovars generally exhibited lower overall enzyme repertoires, particularly pv. pruni, which displayed a reduced amylolytic enzyme profile and showed no amylolytic activity.

Regarding cellulolytic enzymes, the highest average counts were observed in G1, followed by G2 and Xaj; whereas pectinolytic enzymes had the highest numbers in G1, followed by G2. Furthermore, when studying the three main pathovars against G1 and G2, it was observed that these groups harboured not only different numbers but also differential enzymatic profiles. Cellulolytic and pectinolytic enzymes play a fundamental role in bacterial endophytic lifestyle, assisting the bacteria in colonizing, invading and spreading within plant host tissues [91]. Especially, pectinolytic enzymes may play a key role in this process, as pectins are the major matrix polysaccharides in dicotyledonous plants [67], which are the principal hosts of isolation of *X. arboricola* strains. This would also support the higher content of such enzymes in the low-, non-pathogenic strains in G1 and G2.

Conversely, the strains in the three main pathovars showed a lower amount of pectinolytic enzymes, which correlated with their inability to cause soft rot in pepper fruits, suggesting a direct relationship between pectinolytic enzyme abundance and maceration capacity. Furthermore, soft rot has been attributed to pectinolytic activity in *Xanthomonas* due to the evidence that only pectinolytic *Xanthomonas* strains have been shown to cause tissue maceration in vegetables [53,92]. Consequently, the soft rot test is regarded as a reliable indicator of pectinolytic activity [62] and, as such, has been considered in this work. However, functional experiments should be conducted to definitively conclude this association and, more importantly, to determine if there are specific pectinolytic enzymes involved, such as AAM39441.1, which is G1 and G2-exclusive. Therefore, considering the soft rot test as an indicator of pectinolytic activity, the present results were consistent with those from Zarei *et al*., who showed that Iranian *X. arboricola* strains belonging to pvs. pruni and juglandis lacked pectinolytic activity, while strains that did not belong to any of the three main pathovars showed this activity [93].

We hypothesize that strains in G1 and G2 act as opportunistic pathogens, meaning they can infect plant hosts primarily when predisposed by abiotic and/or biotic stress conditions [57,94]. These strains likely exhibit a commensal lifestyle under normal conditions but may shift to a pathogenic state under certain circumstances, such as on damaged leaves or in conditions associated with reduced host defence capacity. In G1 strains, their reduced T3Es repertoire may contribute to their conditional pathogenicity, whereas the putative virulence of G2 appears to be associated with CWDE. Additional putative virulence factors in these groups remain to be investigated in order to better understand the causes of the putative diseases attributed to them. Several strains in G1 and G2 have exhibited doubtful, atypical or uncertain pathogenicity consistent with an opportunistic lifestyle as observed in low-pathogenic Australian tomato isolates mentioned before and in *X. arboricola* pv. celebensis strains, which cause atypical symptoms, such as rotting of the rhizome and fruit and vascular spread [6,57]. More notably, the virulence of *X. arboricola* pv. fragariae strains, which belong to both G1 and G2, has been widely debated, with some authors claiming it as a primary pathogen, while others defining it as a saprophytic or an opportunistic pathogen [5,95]. While initial studies reported symptoms of bacterial leaf blight in strawberry caused by pv. fragariae, subsequent work often failed to reproduce these results [7,8, 55, 62]. However, later studies confirmed symptom development in specific cultivars under high humidity, indicating that factors, such as environmental conditions and pectinolytic activity may influence disease expression [95]. Given that high-humidity conditions are known to promote host colonization by opportunistic or weak pathogens [96], the high humidity required for *X. arboricola* pv. fragariae for developing infections would support its nature as an opportunistic rather than a primary pathogen. In addition, the data from the present study showed that *X. arboricola* pv. fragariae is genetically diverse, with no pv. grouping of the strains as observed in *Xap*, *Xaj* and *Xac*, which had also been described in previous research [5,54, 62]. Furthermore, the lack of recent reports of significant crop losses supports its characterization as a weak or non-aggressive pathogen [62,97, 98]. Importantly, *X. arboricola* pv. fragariae has sometimes been co-isolated with the strawberry pathogen *X. fragariae* [98]. Likewise, Sadhukhan *et al*. observed that co-infection of *X. perforans* with weakly pathogenic strains of *X. arboricola* and *Pseudomonas capsica* caused higher disease severity on tomato and higher growth of the two weakly pathogenic strains. Their study also suggested certain potential synergism among *X. perforans* and *X. arboricola* under high-humidity conditions, probably due to synchronized behaviour and expression of T3Es or CWDE [96]. More research should be done to elucidate whether similar interactions may occur between *X. fragariae* and *X. arboricola* pv. fragariae. Moreover, Pfeilmeier *et al*. showed that the opportunistic *X. hortorum* Leaf131 caused dysbiosis and lowered fresh weight in immunocompromised *A. thaliana* inoculated with a synthetic bacterial community containing this strain. The main virulence factor involved in the formation of the dysbiotic community was the T2SS Xps, which secreted CWDE that would liberate nutrients from the host tissue, benefiting Leaf 131 and the commensal strains in the community [92]. These data suggest that opportunistic *Xanthomonas* may play a role in ecological relationships with pathogenic and commensal strains, contributing to virulence via CWDE, especially in immunocompromised hosts. However, regardless of the putative opportunistic nature of strains in G1 and G2, previous studies showed a certain degree of specificity. For instance, Ferrante and Scortichini [95] assessed host specificity by infiltrating *X. arboricola* pv. fragariae into strawberry *cv*. Candonga and in non-host species, as well as inoculating *Xaj* and *Xac* on strawberry, and measuring bacterial populations via plate counts. Only *X. arboricola* pv. fragariae maintained high populations in strawberries, while numbers declined in the rest of the plant-pathogen combinations, which suggests a pathogenic and specific relationship towards its host [95]. The factors determining the specificity, if any, of strains in G1 and G2, particularly those belonging to less relevant pathovars like pv. fragariae, remain to be elucidated.

In line with the observations discussed above, the pathogenic nature of the strains associated with pathovars *Xap*, *Xac* and *Xaj* seems to be well-established and consistently supported by genomic and experimental evidence. In contrast, strains belonging to groups G1 and G2, whether or not assigned to a specific pathovar, exhibit uncertain or inconsistent pathogenic potential. Among those strains of G1 and G2 for which pathogenicity has been reported, none have been associated with the development of significant outbreaks or epidemics. These strains have been variably referred to in the literature using terms, such as ‘look-alike a pathogenic group’ or ‘weakly pathogenic strains,’ which may lead to misinterpretation or the erroneous assumption of pathogenicity. In certain cases, such as strain CITA 44, pathogenicity has been consistently disproven, while in others, observed reactions appear to correspond to hypersensitive responses dependent on the host genotype. Given the lack of comprehensive pathogenicity assays for all strains included in this study, we propose discontinuing the use of ambiguous designations within the species *X. arboricola*. Instead, we recommend classifying strains strictly as pathogenic or non-pathogenic, based exclusively on robust pathogenicity evidence. All other strains should be considered non-pathogenic until proven otherwise on a compatible host.

This study provides a comprehensive genomic analysis of *X. arboricola*, encompassing both pathogenic and non-pathogenic strains, to better understand its ecological diversity. The results suggest distinct lifestyles across strain groups, ranging from pathogenic to opportunistic or commensal behaviour, highlighting the complexity of host–microbe interactions. Further research is needed to elucidate the molecular mechanisms underlying these lifestyles, as well as the influence of environmental and host-related factors. Such knowledge is essential for improving disease management, enabling more accurate strain differentiation and supporting the development of targeted and sustainable control strategies.

## Supplementary material

10.1099/mgen.0.001782 Supplementary Material 1.

10.1099/mgen.0.001782 Supplementary Material 2.

## Abbreviations

ANI: average nucleotide identity
BAN: brown apical necrosis
CDD: Conserved Domain Database
CWDE: cell wall-degrading enzymes
GH: glycoside hydrolases
NCBI: National Center for Biotechnology Information
OMV: outer membrane vesicle
TALEs: transcription activator-like effectors
T3Es: type III effectors
T2SS: type II secretion system
T3SS: type III secretion system
VOC: vertical oozing canker
WBB: walnut bacterial blight
