# Supplementary material for: Genomic and enzymatic signatures underlying lifestyle diversity in Xanthomonas arboricola
Source: Microb Genom. 2026 Jul 16;12(7):001782. doi: 10.1099/mgen.0.001782 (PMC13375143; doi:10.1099/mgen.0.001782)

Supplementary Figure 1. ANI heatmap including the genomes of all the *Xanthomonas arboricola* strains deposited in GenBank by March 2025. Xap = *X. arboricola* pv. pruni; Xaj = *X. arboricola* pv. juglandis; Xac = *X. arboricola* pv. corylina.

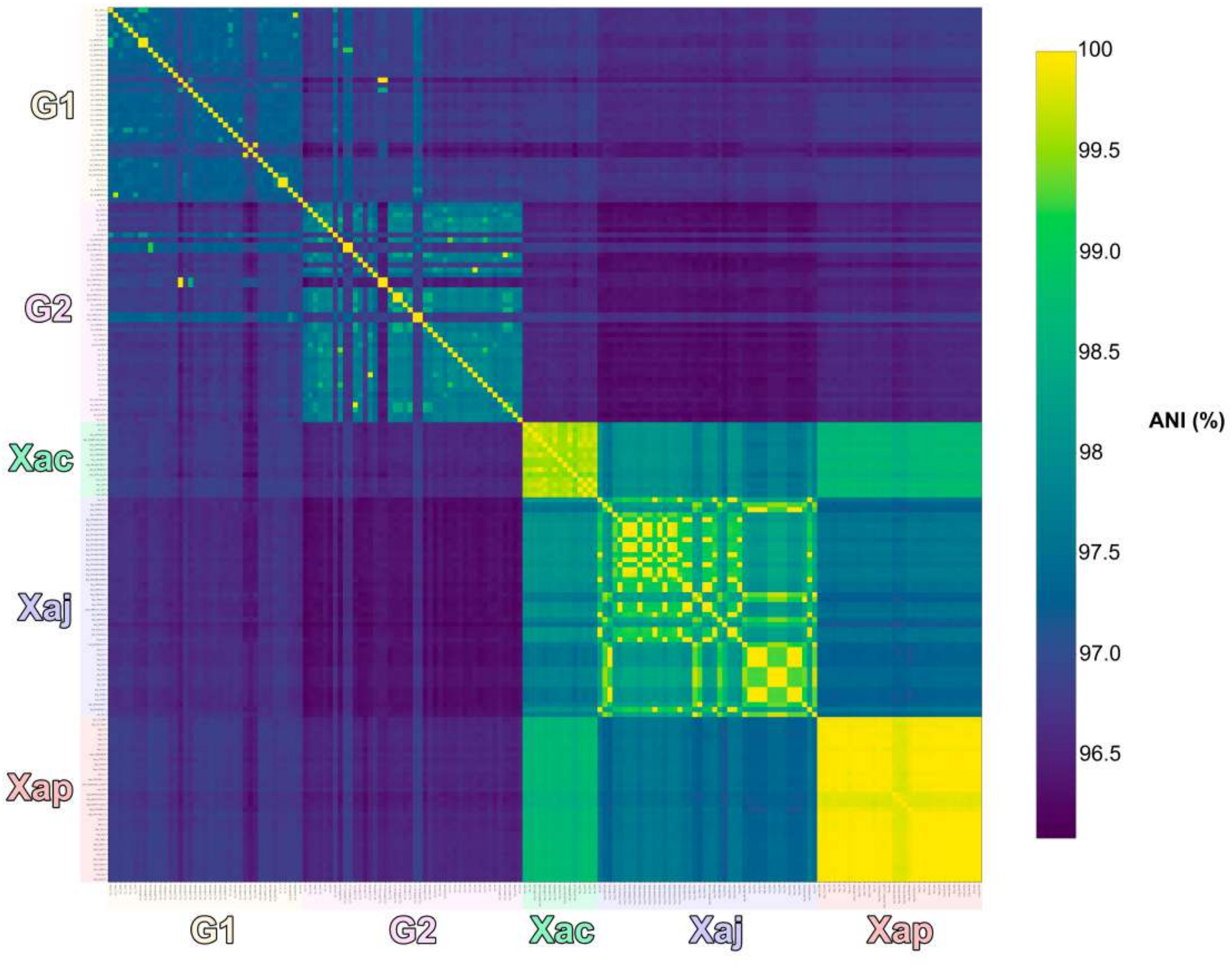

Supplement: Supplementary Material 1. [file mgen-12-01782-s001.pdf]
